# Primary colorectal small cell carcinoma: A clinicopathological and immunohistochemical study of 10 cases

**DOI:** 10.1186/1746-1596-2-35

**Published:** 2007-09-05

**Authors:** Dina El Demellawy, Mahmoud A Khalifa, Nadia Ismiil, Shun Wong, Zeina Ghorab

**Affiliations:** 1 Department of Pathology, Thunder Bay Regional Health Sciences Center, 1874 Oliver Road, Thunder Bay, Ontario P7G 1P1, Canada; 2 Department of Pathology, Sunnybrook Health Sciences Center, 2075 Bayview Avenue, Toronto, Ontario M4N 3M5, Canada; 3 Department of Radiation Oncology, Sunnybrook Health Sciences Center, 2075 Bayview Avenue, Toronto, Ontario M4N 3M5, Canada

## Abstract

Colorectal small cell carcinoma (SmCC) is a rare tumor with an aggressive course. The aim of this study is to summarize our experience with this tumor and to highlight its immunohistochemical profile. Ten cases of colorectal SmCC were identified in our files and a panel of immunostains was performed. Follow up was available for the average of 3 years, during which 7 patients died and 3 were alive with disease. All cases were positive for LMWK, CK 19 and pancytokeratin but were negative for TTF-1 and CA 125. EGFR was positive in 7 cases. TTF-1 negative staining may be valuable in differentiating it from its pulmonary counterpart. CDX2, mCEA, CD56, synaptophysin, NSE and chromogranin can help differentiate it from non-endocrine poorly differentiated adenocarcinoma. The expression of EGFR in a subset of patients has not been reported earlier and has to be evaluated in larger series to assess its role in the planning of targeted biologic therapy.

## Background

Primary extra-pulmonary small cell carcinoma (SmCC) is a rare tumor encountered in several organ systems. In the digestive system, neuroendocrine carcinomas comprise less than 1% of all colorectal cancer and are classically associated with poor outcome [1]. In the few reports of colorectal SmCC (CRSmCC) in the English literature, these tumors show a similar demographic characteristics to colorectal adenocarcinomas with the exception of being more common in females. Because of the difference in prognosis and management between the two tumor types, the distinguishing histological and immunophenotypic features need to be well characterized. The aim of this study is to describe a single institution's experience with this rare tumor, focusing on its immunohistochemical features and possible utilization.

## Materials and methods

This study was approved by the Institutional Research Ethics Board. Since a relatively new laboratory information system was installed in 1999, we were able to search the Anatomic Pathology database and the electronic patients' charts, between 1999 and 2006 for CRSmCC in Sunnybrook Health Sciences Center. Demographic information, clinical data, tumor stage, treatment and follow up were reviewed. Patients with colorectal well-differentiated neuroendocrine tumors (carcinoid tumor), large cell neuroendocrine carcinoma, poorly differentiated carcinoma with focal neuroendocrine differentiation or with history of Merkel cell tumors of the skin, primary SmCC of the lung or other organs were excluded. All patients included in this study had either chest plain radiograph or a chest CT that was free of tumor.

**Figure 1:**
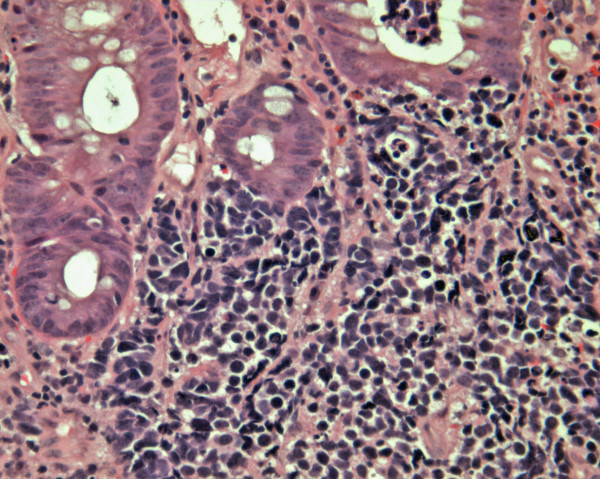
A colonic small cell carcinoma with brisk mitoses, apoptosis and necrosis. Adjacent colonic mucosal crypts are non-dysplastic. (H&E × 400).

H&E-stained sections were reviewed by two pathologists to confirm the diagnosis. The histological criteria applied for the diagnosis were the same as those used for the diagnosis of pulmonary SmCC. These criteria required that the entire tumor in the submitted material consists of cohesive round to oval cells with sparse cytoplasm, nuclei with salt and pepper chromatin, nuclear moulding and small or inconspicuous nucleoli. These features needed to be present in the setting of brisk mitoses, apoptosis and necrosis (Figure 1). As per the local surgical pathology protocols, submitted representative tissue sections were routinely processed and embedded in paraffin. Sections (2–3 μm) of the tumor, through its most invasive part and including adjacent normal mucosa when possible, were stained for immunohistochemistry using standard avidin-biotin complex method. The panel of antibodies used summarized in Table 1. Results of immunostains were assessed by two pathologists and a consensus regarding controversial cases was reached at the double-headed microscope. Evaluation of the immunohistochemical staining was performed by light microscopy using a 10× eye-piece lense with the selective use of 20 – 40× objective lense for confirmation. The markers were evaluated by a semiquantitative method. With the exception of CDX2 and TTF-1 which showed nuclear staining, and EGFR which showed cell membrane staining, cytoplasmic staining was required for all other immunostains.

**Table 1:** The panel of immunohistochemical reagents used

**Antibody**	**Clone**	**Manufacturer**	**Pre-treatment**	**Dilution**
CK 7	OV-TL 12/30	Dako (Carpinteria, CA)	Pepsin for 10 minutes at 37°C	1/4000
CK 20	KS 20.8	Novocastra (Vision Biosystem, Norwell, MA)	Pepsin for 10 minutes at 37°C	1/50
Synaptophysin	27G12	Vector (Burlingame, CA)	HIER at pH 6.0	1/200
Chromogranin A	Polyclonal	Dako (Carpinteria, CA)	Pepsin for 10 minutes at 37°C	1/200
CD56	123C3	Zymed (Invitrogen, Carlsbad, CA)	HIER at pH 6.0	1/200
NSE	Polyclonal	Dako (Carpinteria, CA)	None	1/250
EGFR	31G7	Zymed (Invitrogen, Carlsbad, CA)	Pepsin for 10 minutes at 37°C	1/100
LMWK	Cam 5.2	B.D. Bioscience (San Jose, CA)	Pepsin for 10 minutes at 37°C	1/64
CK 19	6170	Novocastra (Vision Biosystem, Norwell, MA)	Pepsin for 10 minutes at 37°C	1/50
Pankeratin	AE1/AE3	Dako (Carpinteria, CA)	Pepsin for 10 minutes at 37°C	1/300
TTF-1	8G7G3/1	Neomarker (Lab Vision, Fremont, CA)	HIER at pH 6.0	1/800
CA 125	Ov185:1	Novocastra (Vision Biosystem, Norwell, MA)	HIER at pH 6.0	1:100
CDX2	Amt 28	Vision Biosystem (Norwell, MA)	HIER at pH 8.0	1/50
mCEA	B80-1	Biomeda (Foster City, CA)	None	1/200

HIER = Heat-induced epitope retrieval, in a Biocare decloaking chamber

## Results

Ten patients with primary CRSmCC were identified with their demographic and clinical findings summarized in Table 2. Their age ranged from 39 – 88 (median = 60). There were 7 females and 3 males. Family history of colorectal cancer was present in one patient and 2 had a history of inflammatory bowel disease. Three patients had synchronous adenomas in the large bowel. Nine cases presented with symptoms related to a colorectal mass and/or metastasis while one case presented with biliary colic. Seven cases had tumors arising in the rectum including the anorectal junction. Nine of the ten patients had distant metastases at the time of diagnosis. The commonest metastatic site was the liver present in 6 cases.

**Table 2:** Demographic and clinical summary

Case	Age/Sex	Site	History	Present-Ation	Metastasis/Site	Procedure	Associated Pathology	Follow up
1	65/M	Ileocecal	? Cholecystitis	Abdom-inal Pain	-	Right Hemicolectomy (pT3 N2)	Tubulovillous adenoma with invasive adenocarcinoma adjacent to SmCC	DOD
2	69/M	Ascending colon	Family history of colon cancer	Abdominal pain	+/Liver	Biopsy + Right Hemicolectomy (pT2 N2)	Adenoma	DOD
3	88/F	Ascending colon	Mass adjacent to colon	Pleural Effusion	+/Medias-tinal lymph nodes and liver	Biopsy	-	DOD
4	42/F	Rectum	2 previous negative rectal biopsies a year earlier	Abdominal pain	+/Liver	Biopsy	-	AWD
5	43/F	Rectum	Ulcerative colitis	Abdominal Pain and weight loss	+/Retro-peritoneal, inguinal lymph nodes, liver and skin	Biopsy	Ulcerative colitis	DOD
6	49/F	Rectum	Rectal villous adenoma 2 years earlier	Abdominal Pain	+/Para-aortic lymph nodes and liver	Biopsy	-	DOD
7	59/F	Rectum	Crohn's disease	Abdominal pain	+/Para-aortic lymph nodes	Biopsy + Low anterior resection (pT3 N2)	Crohn's disease + 3 tubular adenomas away from SmCC	DOD
8	66/F	Rectum	-	Stool incontinence + Rectal pain	+/Groin lymph nodes	Biopsy	-	AWD
9	39/M	Rectum	-	Rectal urgency	+/Exten-sion to pelvic side walls	Biopsy, loop colostomy, then abdomino-perineal resection	SIADH	DOD
10	61/F	Ano-rectal junction	-	Rectal bleeding	+/Liver	Biopsy	-	AWD

AWD = Alive with disease. DOD = Dead of disease

On routine H&E sections, all our cases showed solid nests and clusters of small to medium sized cells, displaying prominent apoptosis, nuclear moulding, brisk mitosis (>10/10 HPFs) and necrotic foci. Their nuclei showed classic salt and pepper chromatin with indistinct nucleoli. Occasional rosette formation was noted. Two of the cases were associated with other colonic pathology; one of which showed a colonic adenoma away from the SmCC while the other showed colonic adenocarcinoma arising from tubulovillous adenomas, merging with the lateral edge of the SmCC. In all the cases, there was no evidence of divergent differentiation in tehe form of squamous, acinar (glandular) or large cell neuroendocrine. Lymphovascular invasion was present in all of our cases. None of the cases showed Azzopardi effect, however crushing artefacts were noted in all the cases.

**Figure 2:**
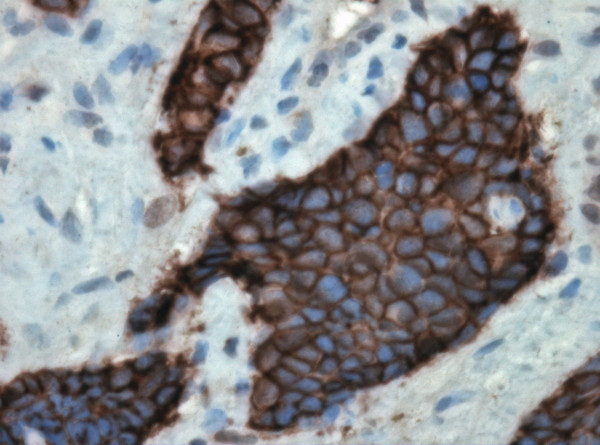
A small cell carcinoma of the ascending colon with immunopositivity to CD56. (CD56 × 400).

**Figure 3:**
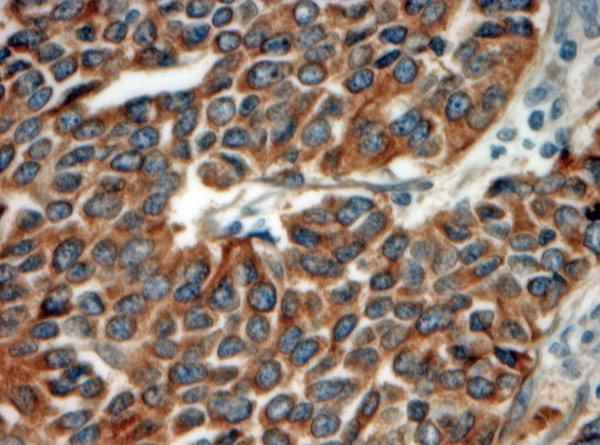
A rectal small cell carcinoma showing positive synaptophysin immunostaining. (Synaptophysin × 400).

**Figure 4:**
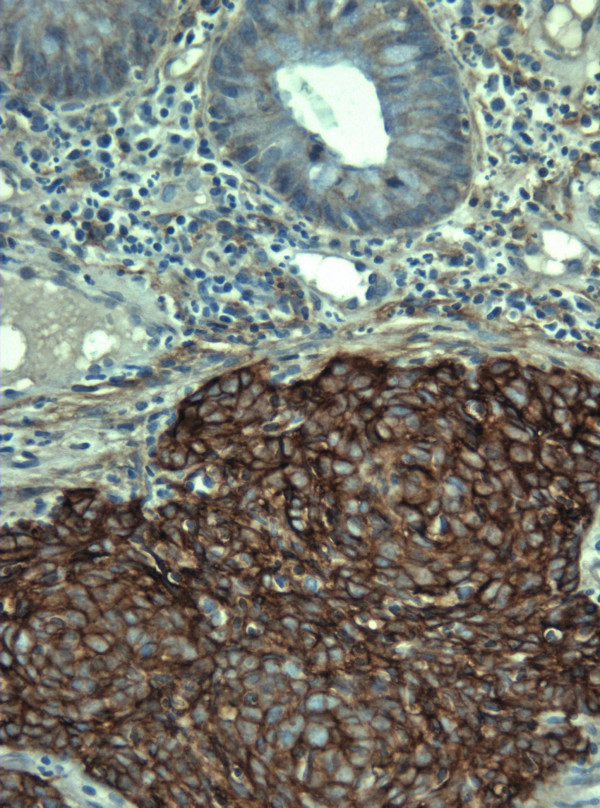
A rectal small cell carcinoma showing positive EGFR immunostaining. (EGFR × 400).

Our immunohistochemical panel showed that all cases were positive for low molecular weight cytokeratin (LMWK), CK 19 and pancytokeratin but were negative for TTF-1 and CA 125. CK 7 expression was variable but that of CK 20 was restricted to tumors of the right colon. Immunostaining for neuroendocrine differentiation including CD56, synaptophysin, NSE, and chromogranin was variable but every case showed positivity with at least one of these markers (Figures 2 and 3). CDX2 was positive in only 2 cases while mCEA was positive in 2 cases. EGFR was positive in 7 cases (Figure 4). Results of the immunohistochemistry are summarized in Table 3. The case with the associating adenocarcinoma component showed similar immunohistochemical profile (including EGFR expression) in both tumors, except for the lack of CD56, synaptophysin and chromogranin expressions and the presence of CEA expression in the adenocarcinoma.

**Table 3:** Summary of immunohistochemical staining

Case	CK 7	CK 20	Synp	Chrom	CD 56	NSE	EGFR	LMWK	CK 19	AE1/AE3	TTF-1	CA 125	CDX 2	mCEA
1	-	+	+	+	+	+	+	+	+	+	-	-	-	-
2	-	+	+	-	+	+	-	+	+	+	-	-	-	+
3	+	-	-	-	+	-	+	+	+	+	-	-	-	-
4	-	-	+	-	+	+	-	+	+	+	-	-	-	-
5	+	-	-	-	+	-	+	+	+	+	-	-	-	+
6	+	-	-	+	-	+	+	+	+	+	-	-	-	-
7	-	-	+	+	+	-	+	+	+	+	-	-	-	-
8	-	-	+	+	+	+	-	+	+	+	-	-	-	-
9	+	-	+	+	+	+	+	+	+	+	-	-	+	-
10	-	-	+	+	+	+	+	+	+	+	-	-	+	-

Synp = Synaptophysin

Chrom = Chromogranin

The standard management for CRSmCC during this period of review was combined chemotherapy and radiotherapy similar to protocols used in SmCC of the lung.

Five patients received VP16 and cis-platinum with or without consolidation therapy to the local disease. However, two patients deteriorated rapidly and died of metastatic disease prior to the initiation of systemic chemotherapy. Four cases were treated by surgery (2 with right hemicolectomy, 1 with low anterior resection and 1 with abdomino-perineal resection) with or without post-operative adjuvant chemotherapy.

The median follow up was 3 years (10 months – 4 years), during which 7 patients died of disease. The remaining 3 cases were alive with disease at last follow-up.

## Discussion

Primary extra-pulmonary SmCC is uncommon, occurring in only 4% of all patients with SmCC [2]. While its actual incidence may be underestimated, SmCC presents 0.1% to 1% of all gastrointestinal (GI) malignancies, with varying incidence in different organs [3]. SmCC is estimated to represent 1% to 2.8% of all esophageal cancer, with the esophagus being the commonest site for GI SmCC [4-9]. The second most common location is the colorectum where SmCC accounts for 0.2%–0.8% of all colorectal neoplasms [10-12]. Primary SmCC has been described in most organs of the body [3,13-20], except the central nervous system.12 The majority of patients with GI SmCC present with overt distant metastases [3]. In our study, the commonest symptom was abdominal pain and 9 of our 10 cases had metastatic disease at the time of diagnosis. The commonest primary site is the rectum, followed by the cecum and sigmoid colon [3]. Similarly, in our series, the tumor occurred in the rectum including the anorectal junction in 7 patients. In one series, a predominance of the disease in older men (mean age = 64 years) was reported [3]. Our patients ranged in age from 39–88 years old with a median age of 60 years and showed a female predominance with a female to male ratio of 2:1. Three of our 10 cases had adenomas in the vicinity of their tumors and 2 had inflammatory bowel disease but none was immunodeficient. The association of CRSmCC with inflammatory bowel disease and adenoma either within or away from the SmCC has been documented earlier [10,21], however their possible role in CRSmCC carcinogenesis remains uncertain.

The role of immunohistochemistry in establishing the diagnosis of CRSmCC has been a subject of considerable debate. To some authors, the classic histological features on H&E-stained sections are sufficient for the diagnosis [22]. However, for many others, the documentation of neuroendocrine differentiation is essential to compliment the characteristic morphologic features [23]. SmCC usually shows positive staining for chromogranin and synaptophysin with strong and diffuse immunoreactivity for CD56 [22,24]. In our series, CD56 was more sensitive than chromogranin and synaptophysin as it was positive in 9 of the 10 cases. In the current study, we excluded other neuroendocrine tumors as carcinoid, atypical carcinoid and large cell neuroendocrine carcinomas based on tumor morphology in order to limit our immunohistochemical assessment to pure small cell carcinomas.

Pancytokeratin (AE1/AE3) and LMWK (Cam5.2) are reported to be positive in all SmCC [22]. Similar findings were present in our cases and in addition, CK 7 and 19 expression was also noted in all cases. We also report the patterns of staining for cytokeratins 7 and 20 staining with CK 7+/CK 20- in 4 cases, CK 7-/CK 20+ in 2 cases, and CK 7-/CK 20- in 4 cases. Our series is the first to document the limitation of CK 20 immunohistochemistry in CRSmCC. None of the rectal SmCC was positive for CK 20. CK 19 was positive in all 10 cases studied. Since its expression is also noted in conventional colorectal adenocarcinoma, CK 19 has a limited value in distinguishing it from CRSmCC [25]. To the best of our knowledge, there are no previous reports in the English literature describing the expression of cytokeratins 7, 19, and 20 in CRSmCC.

Thyroid transcription factor-1 (TTF-1) is a nuclear homeodomain transcription factor that is expressed in the developing thyroid, respiratory epithelium, and diencephalon [26]. Several studies have documented that TTF-1 could not be used in distinguishing pulmonary from extra-pulmonary SmCC due to the extensive overlap in these tumors' immunophenotypes. With the exception of cases with skin tumors, TTF-1 could not be reliably used to distinguish primary from metastatic SmCC in extra-pulmonary sites [26-28]. In one study, TTF-1 was reported to be positive in the vast majority of pulmonary (81%) and extra-pulmonary (80%) SmCC with the exception of Merkel cell tumor [28]. Another study reported that TTF-1 was expressed in 82.7% of pulmonary SmCCs, 42.0% of extra-pulmonary SmCCs (range, 33.3 – 53.3% for the various sites), and 0% of Merkel cell carcinomas [26]. Interestingly, none of these extra-pulmonary SmCC cases included in both studies involved the colorectum [26,28]. With relatively limited data, staining for TTF-1 seems to be uniformly negative in CRSmCC.[29] The results of our study support this observation highlighting the value of TTF-1 immunostaining in differentiating SmCC of a colorectal from that of a pulmonary origin. This is particularly significant in CRSmCC with lung metastases to exclude primary lung tumors or in patients with synchronous malignancies. However the presence of CK 20 +/TTF-1 – subset of CRSmCC highlights the morphological and immunohistochemical overlap between CRSmCC and Merkel cell carcinoma. CDX2 and mCEA are rarely expressed in CRSmCC and hence they may be of value in differentiating it from poorly differentiated colorectal adenocarcinoma. To the best of our knowledge, no previous study has described CDX2 expression in CRSmCC. The diagnostic value of CDX2 in Merkel cell tumor has not yet been established.

Seven of our cases showed EGFR expression. Reporting EGFR immunostaining in conventional primary colorectal adenocarcinoma and its metastasis has extensively been studied recently [30,31]. The current study is the first to report EGFR expression in CRSmCC which could have management and prognostic implications through the possible use of targeted biological therapy in these tumors, if similar results are shown in larger series.

Similar to SmCC of the lung, CRSmCC usually presents with distant metastases [32-34]. Nine of our 10 patients had metastases at the time of diagnosis. The prognosis of CRSmCC is worse than that of stage-matched conventional colorectal adenocarcinomas [22]. With the exception of Merkel cell tumor, the standard management for SmCC has increasingly become systemic chemotherapy [35] whether the tumor is pulmonary or extrapulmonary. In spite of the initial response to chemotherapy, radiotherapy and possibly surgery depending on the extent of residual disease or response [12,16], patients invariably relapse and die rapidly of distant metastases.

## Conclusion

SmCC is an aggressive tumor of the large bowel with a predilection for the rectum. This study reports for the first time the various CK7/CK20 immunostaining patterns encountered, its predominant negative staining with CDX2 and the expression of EGFR in more than half of these tumors. It elaborates on the practical use of immunohistochemistry in the differential diagnosis.
